# DeepRelaxo: Fast Mono‐Exponential Magnitude Brain R2* Mapping With Reduced Echoes Using Self‐Supervised Deep Learning

**DOI:** 10.1002/mrm.70405

**Published:** 2026-04-26

**Authors:** Samiha Prima, Zhuang Xiong, Alan H. Wilman, Hongfu Sun

**Affiliations:** ^1^ School of Electrical Engineering and Computer Science University of Queensland Brisbane Queensland Australia; ^2^ Image X Institute, Sydney School of Health Sciences University of Sydney Sydney New South Wales Australia; ^3^ Department of Biomedical Engineering University of Alberta Edmonton Alberta Canada; ^4^ School of Engineering University of Newcastle Newcastle New South Wales Australia

**Keywords:** deep learning, DeepRelaxo, Echo reduction, multi‐echo gradient echo (ME‐GRE), R2* mapping, transformer

## Abstract

**Purpose:**

We introduce DeepRelaxo, a fast and generalizable deep learning method for estimating brain R2* maps from multi‐echo gradient echo (ME‐GRE) acquisitions with arbitrary echo configurations, including shortened echo trains for accelerated scans.

**Methods:**

DeepRelaxo is a cascaded two‐stage self‐supervised network comprising: (1) a voxel‐wise Transformer‐MLP for initial R2* estimation, and (2) a patch‐based 3D U‐Net for denoising. Both stages are trained entirely on synthetic ME‐GRE data simulated at 3 T with a varied number of echoes, echo times, and noise levels. We evaluate on simulated and in vivo brain datasets, comparing it against conventional non‐linear least squares (NLLS) and the standalone Transformer‐MLP. Experiments assess robustness under increased noise and shortened TEs.

**Results:**

In simulations, DeepRelaxo consistently outperforms NLLS and Transformer‐MLP, particularly in accelerated conditions. For example, with 4× scan time reduction at low SNR (= 10), DeepRelaxo improves SSIM by 13.5% and reduces RMSE by 76% compared with baseline methods. In in vivo 3 T and 7 T data, DeepRelaxo produces consistent R2* values in deep gray matter and preserves anatomical detail, even with only two short echoes.

**Conclusion:**

DeepRelaxo effectively models ME‐GRE decay, leveraging temporal and spatial context to deliver accurate, robust, and computationally efficient R2* mapping. It enables reliable reconstruction under accelerated protocols, making it suitable for time‐sensitive workflows.

## 1 Introduction

The effective transverse relaxation rate [1] ($R_{2}^{*}=1/T_{2}^{*}$) is particularly sensitive to microscopic magnetic field inhomogeneities caused by local variations in iron content, myelin density, and microvascular architecture [2, 3]. As such, $R_{2}^{*}$ serves as a robust biomarker for assessing iron deposition in neurodegenerative diseases [4] and is frequently used to probe tissue composition in deep gray matter regions [5, 6, 7]. In clinical and research settings, $R_{2}^{*}$ is commonly estimated from multi‐echo gradient‐recalled echo (ME‐GRE) acquisitions, which sample signal decay at multiple echo times. A voxel‐wise fitting to a mono‐exponential model is generally used for $R_{2}^{*}$ mapping reconstruction such as the non‐linear least squares (NLLS) method. However, NLLS fitting is computationally expensive and is particularly susceptible to Rician noise–induced bias, especially in low signal‐to‐noise ratio (SNR) regimes.

To address these limitations, recent advances in quantitative $R_{2}^{*}$ mapping have increasingly leveraged deep learning (DL) to enable efficient and robust reconstruction from noisy and undersampled ME‐GRE acquisitions [8, 9, 10, 11, 12, 13, 14, 15]. Several DL methods integrate domain‐specific priors, such as the biophysical relaxation models within convolutional neural networks (CNNs) [16], including RoaR [12] and LEARN‐BIO [14]. Complementary lines of work address reconstruction from undersampled k‐space [15, 17, 18], including qRIM [15], CoRREct [13], PHIMO+ [18]. Beyond brain imaging, deep learning‐based models have also been applied to liver [9, 10, 11], including UP‐Net [10] and CadamNet [9].

Despite these advances, practical deployment of DL‐based R2* mapping remains challenging. ME‐GRE acquisitions used for R2* mapping differ substantially across scanners and studies, including variations in echo count, echo spacing, and acquisition timing. Such heterogeneity poses a significant generalization challenge for pretrained neural networks that are typically optimized for fixed echo configurations. Furthermore, a major barrier to clinical $R_{2}^{*}$ mapping is long scan time. Accurate estimation typically requires 4–10 echoes with wide spacing and long durations, resulting in 5–10 min acquisitions. Reducing scan time by using fewer, shorter echoes is desirable, especially for patients unable to tolerate lengthy scans. However, most current deep learning‐based R2* mapping algorithms are not designed for such undersampled echo trains and therefore degrade in performance when the available echoes are reduced. An approach that remains accurate under aggressively shortened or heterogeneous echo trains would substantially improve the practicality and clinical feasibility of R2* mapping.

Encouragingly, recent work in DL‐based relaxometry has shown that quantitative parameter mapping remains feasible even under severe temporal undersampling. For example, the DL‐assisted Look–Locker approach [19] achieves accelerated T_1_ mapping from only a few inversion‐recovery samples, while DeepEMC‐T2 [20] models the echo‐modulation curve to enable accurate T_2_ estimation from shortened echo trains. These studies demonstrate that deep learning can compensate for sparse temporal sampling by learning relaxation dynamics. Nevertheless, these methods remain tied to fixed acquisition schemes such as predetermined inversion times or specific echo refocusing trains and therefore do not generalize across heterogeneous protocols.

In this study, we introduce DeepRelaxo, a deep learning framework that addresses these limitations by moving beyond conventional voxel‐based multilayer perceptrons (MLPs) and patch‐based U‐Nets that rely on fixed ME‐GRE acquisition requirements. Built on a Transformer architecture with a cross‐attention mechanism, DeepRelaxo explicitly models the relationship between echo times and signal magnitudes, enabling it to process echo series of arbitrary length and timing. As a result, DeepRelaxo generalizes across heterogeneous ME‐GRE protocols, operating robustly under variable echo counts, arbitrary TE configurations, and multiple echo‐subsampling patterns. The proposed framework maintains high accuracy under high‐noise and limited‐echo conditions while substantially reducing reconstruction time, highlighting its potential for fast, robust, and clinically feasible R2* mapping.

## 2 Methods

### 2.1 $R_{2}^{*}$ Mono‐Exponential Decay Model

The transverse relaxation rate $R_{2}^{*}$, can be estimated from the ME‐GRE signal acquired at each voxel. The ideal, noise free $R_{2}^{*}$ signal decay is generally modeled in a mono‐exponential form [21, 22], while, in practice, the measured signal is affected by an additive noise term:

(1) $$M_{t}=M_{0}\cdot \exp\left(-R_{2}^{*}\cdot t\right)+\varepsilon$$

Here $M_{0}$ is the signal intensity at t=0, $M_{t}$ is the signal measured at time t and ε represents Rician noise [23, 24] present in magnitude data. Note that this model assumes a single‐compartment mono‐exponential decay in magnitude images and does not explicitly account for macroscopic $B_{0}$ field inhomogeneity. By acquiring signals at multiple echo times, a decay curve is formed at each voxel from which $R_{2}^{*}$ can be estimated by the non‐linear least‐squared (NLLS) method:

(2) $$\underset{M_{0},{\;R}_{2}^{*}}{\text{argmin}}\;\sum_{i=1}^{N}{\left[M_{i}-M_{0}\cdot \exp\left(-R_{2}^{*}\cdot t_{i}\right)\right]}^{2}$$

Here N is the number of echoes, $M_{i}$ is the observed signal at echo time $t_{i}$. Different curve fitting algorithms can be used to solve this optimization, such as MATLAB's “fmincon” function. This fitting is performed voxel‐wise, often in parallel across slices for efficiency.

The accuracy of this fitting depends on acquisition parameters, such as the distribution of echo times and corresponding signal intensities, which collectively influence the stability and accuracy.

### 2.2 DeepRelaxo Cascaded Neural Networks

**FIGURE 1:**
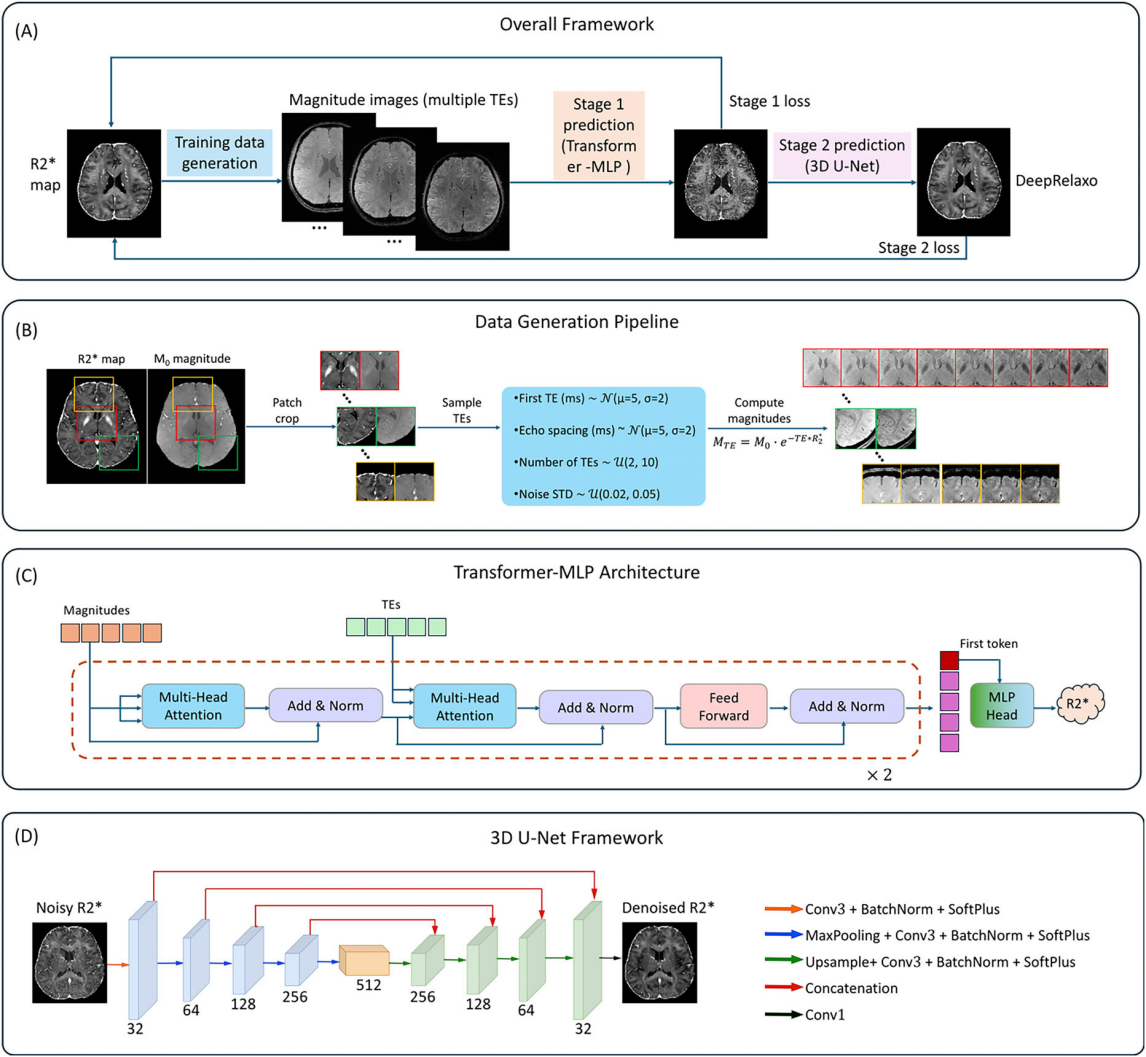
(A) Overview of data generation and the two‐stage training pipeline. (B) Illustration of synthetic data generation using randomly sampled acquisition parameters, including echo times and noise levels. (C) Decoder‐based Transformer‐MLP architecture with cross‐attention mechanism. (D) 3D U‐Net Denoiser architecture with encoder and decoder blocks.

As illustrated in Figure 1A, the proposed DeepRelaxo consists of two sequential modules: (i) a voxel‐based Transformer‐MLP $R_{2}^{*}$ estimation module that models signal‐time relationships using the cross‐attention mechanism [25], (ii) a patch‐based 3D U‐Net that incorporates spatial context to refine and denoise the initial predictions, which is particularly powerful for shorter echo trains. The following sections describe each module in detail. Codes are available at: https://github.com/sunhongfu/DeepRelaxo.

#### 2.2.1 DeepRelaxo Estimator (Transformer‐MLP)

The first stage of DeepRelaxo is a Transformer‐MLP model, which predicts voxel‐wise R2* from ME‐GRE magnitude sequences $M=\left[M_{1},M_{2},M_{3},\ldots M_{j},\ldots \right]$ and echo times $t=\left[t_{1},t_{2},t_{3},\ldots t_{j},\ldots \right]$:

(3) $${\hat{R_{2}^{*}}}^{\left(i\right)}=A_{\theta }\left(M^{\left(i\right)},t\right),$$

where $A_{\theta }$ denotes the Transformer‐MLP network parameterized by learnable weights θ, and ${\hat{R_{2}^{*}}}^{\left(i\right)}$ denotes the estimated R2* value of voxel i.

To train this model, we generated 8 million synthetic voxel samples, as illustrated in Figure 1B. For each voxel sample, a ground‐truth R2* was drawn uniformly from $\left[0,200\right]\;s^{-1}$. The number of echoes was randomly selected from 2 to 10, with the first echo time and inter‐echo spacing sampled from a normal distribution (mean 5 ms, standard deviation 2 ms, bounded within [2, 8] ms). Noise‐free magnitudes were simulated using the exponential decay model, then corrupted with Rician noise by adding Gaussian noise (standard deviation uniformly sampled from [0.02, 0.05]) to the real and imaginary components, as in Equation [1]. Macroscopic B_0_ field inhomogeneity was not modeled in the synthetic data generation used for model training or evaluation. To enable mini‐batch training in parallel, all magnitude and echo‐time sequences were zero‐padded to length 10, with padding tokens set to −999 and excluded from model computations via a padding mask.

Detailed descriptions of the Transformer‐MLP architecture and ablation studies on network parameter selection are provided in the Supporting Information. Training was performed with a mini‐batch size of 200 on an NVIDIA H100 GPU with 80 GB VRAM. Optimization used Adam optimizer ($\beta_{1}=0.5,\beta_{2}=0.999,\epsilon =10^{-9}$, weight decay = $5\times 10^{-4}$) with an initial learning rate 0.001, reduced by a factor of 0.1 every 50 epochs. Transformer‐MLP was trained for 200 epochs using mean squared error loss, with a total training time of approximately 12 h.

#### 2.2.2 DeepRelaxo Denoiser (3D U‐Net)

The second stage of DeepRelaxo is a 3D U‐Net [26] denoiser that refines voxel‐wise R2* estimates (${\hat{R_{2}^{*}}}^{\left(i\right)}$) from the Transformer‐MLP:

(4) $$R_{2}^{*}=F_{\phi }\left({\hat{R_{2}^{*}}}^{\left(1\right)},\cdots ,{\hat{R_{2}^{*}}}^{\left(i\right)},\cdots ,{\hat{R_{2}^{*}}}^{\left(N\right)}\right),$$

where $F_{\phi }$ denotes the 3D U‐Net parameterized by learnable weights ϕ, and N is the total number of voxels of the 3D volume.

A patch‐based synthetic dataset was generated to train the 3D U‐Net Denoiser module of DeepRelaxo. We randomly selected 110 full‐brain $R_{2}^{*}$ and M_0_ maps from an in vivo 3 T ME‐GRE dataset [27] reconstructed by NLLS. Each map was brain‐masked (BET/FSL) and cropped to the smallest bounding box (determined from the nonzero voxel extent of the brain mask) to exclude background and partitioned into patches of size $48^{3}$ using a sliding window with a stride of 20 voxels. This yields a total of 46 200 patches for training. For each patch, the number of echoes, initial echo time, and echo spacing were randomly generated as in voxel‐based data preparation. Signal magnitudes were simulated with Equation [1] and corrupted with Rician noise [24, 28, 29] of standard deviations 0.04, 0.05, and 0.06 relative to the mean signal of the first echo, corresponding to approximate SNRs of 25, 20, and 15.

Each synthetic 3D patch was first processed voxel‐wise by the trained Transformer‐MLP, serving as input to the 3D U‐Net Denoiser. The 3D U‐Net was then trained on these inputs with ground‐truth patch R2* maps as labels. Detailed descriptions of the 3D U‐Net architecture and ablation studies on network parameter selection are provided in the Supporting Information. Training was performed with a mini‐batch size of 100 on an NVIDIA H100 GPU (80 GB VRAM). Optimization used Adam optimizer ($\beta_{1}=0.5,\beta_{2}=0.999,\epsilon =10^{-9}$, weight decay = $5\times 10^{-4}$) with an initial learning rate 0.001, halved every 15 epochs. This 3D U‐Net denoiser was trained for 150 epochs with mean squared error loss, taking ∼17.6 h.

### 2.3 Experiments

#### 2.3.1 Simulated Test Data

We generated a separate set of simulated test participants by forward simulating on five new in vivo ME‐GRE brains as described in Section 2.2.2, using a fixed 8‐echo configuration (first TE = 3.2 ms, spacing = 2.7 ms). For forward simulation, tissue‐specific R2* values were derived from in vivo 3 T ME‐GRE data using traditional voxel‐wise fitting. Mean ± standard deviation R2* values were 19.8 ± 0.8 s^−1^ for WM and 17.7 ± 1.2 s^−1^ for GM consistent with previously reported in vivo measurements at 3 T [30, 31]. Complex Gaussian noise (standard deviation 0.01–0.06) was added to the simulated signals to generate a broad SNR range for robust quantitative evaluation.

#### 2.3.2 In Vivo Acquired Test Data

We conducted four sets of in vivo acquisitions on different scanners of varying acquisition parameters to test the robustness and the generalizability of the DeepRelaxo model. Institutional ethics board approval was obtained, and all subjects gave informed written consent. Prior to model inference, voxel spread function (VSF) correction [32] was applied to the raw ME‐GRE data to compensate for macroscopic B0 field inhomogeneity effects as a pre‐processing step before the R2* mapping process (Figure S3). Details of the in vivo experiments are presented below.

- Participant #1: 27‐year‐old male on a 3 T Siemens Prisma system. Whole‐brain 3D ME‐GRE acquisition with 5 equally spaced unipolar echoes (TEs = 4.9–24.7 ms), 1 mm isotropic spatial resolution, FOV = 256 × 256 × 192 mm^3^, TR = 30 ms, flip angle = 15°, 32‐channel head coil with GRAPPA acceleration factor 3, total scan time 4.3 min.
- Participant #2: 61‐year‐old female on a 3 T Siemens Skyra system. Whole‐brain 3D ME‐GRE acquisition with 9 equally spaced unipolar echoes (TEs = 5.8–44.2 ms), 1 mm isotropic spatial resolution, FOV = 224 × 182 × 144 mm^3^, TR = 50 ms, flip angle = 25°, 32‐channel head coil with GRAPPA acceleration factor 3, total scan time 8.7 min.
- Participant #3: 32‐year‐old male on a 7 T Siemens Terra system. Whole‐brain 3D ME‐GRE acquisition with 9 equally spaced bipolar echoes (TEs = 5.1–21.4 ms), 0.75 mm isotropic spatial resolution, FOV = 210 × 210 × 144 mm^3^, TR = 24 ms, flip angle = 13°, 32‐channel head coil with GRAPPA acceleration factor 2, total scan time 9.5 min.
- Training set cohort: 110 participants on a 3 T GE Discovery 750 system. Whole‐brain 3D ME‐GRE acquisition with 8 unipolar readout echoes (TEs = 3.4–27.9 ms), 1 mm isotropic spatial resolution, FOV = 256 × 256 × 28 mm^3^, TR = 29.8 ms, flip angle = 20°, 12‐channel head coil with ASSET acceleration factor 2, total scan time 5.9 min.
- Reproducibility participants: All scan–rescan data from the three additional participants (27‐year‐old female, 30‐year‐old female, 27‐year‐old male) were acquired using the training set cohort acquisition protocol described above.

### 2.4 Performance Analysis and Methods Comparison

To test robustness under shortened acquisitions, we progressively truncated echoes from the end of the echo train, retaining only earlier echoes to simulate reduced scan times. The minimum case used only the first two echoes. These experiments were conducted on both simulated and in vivo datasets. For simulations, reconstructed R2* maps were evaluated using whole‐brain Structural Similarity Index (SSIM), Root Mean Square Error (RMSE), Peak Signal‐to‐Noise ratio (PSNR), and High‐Frequency Error Norm (HFEN). DeepRelaxo (Transformer‐MLP + 3D U‐Net) was compared against Transformer‐MLP alone and conventional NLLS fitting on five simulated participants.

For in vivo data, reconstructions were assessed qualitatively for tissue contrast and noise suppression under echo reduction. Additionally, region‐of‐interest (ROI) analysis was performed from 10 participants in six deep gray matter structures (Putamen, Caudate, Globus Pallidus, Thalamus, Red Nucleus, Substantia Nigra), where the mean and standard deviation of R2* values were compared across methods and echo settings. Reproducibility was assessed using scan–rescan ROI measurements through Bland–Altman analysis and Pearson correlation.

## 3 Results

### 3.1 Simulation Experiments

**FIGURE 2:**
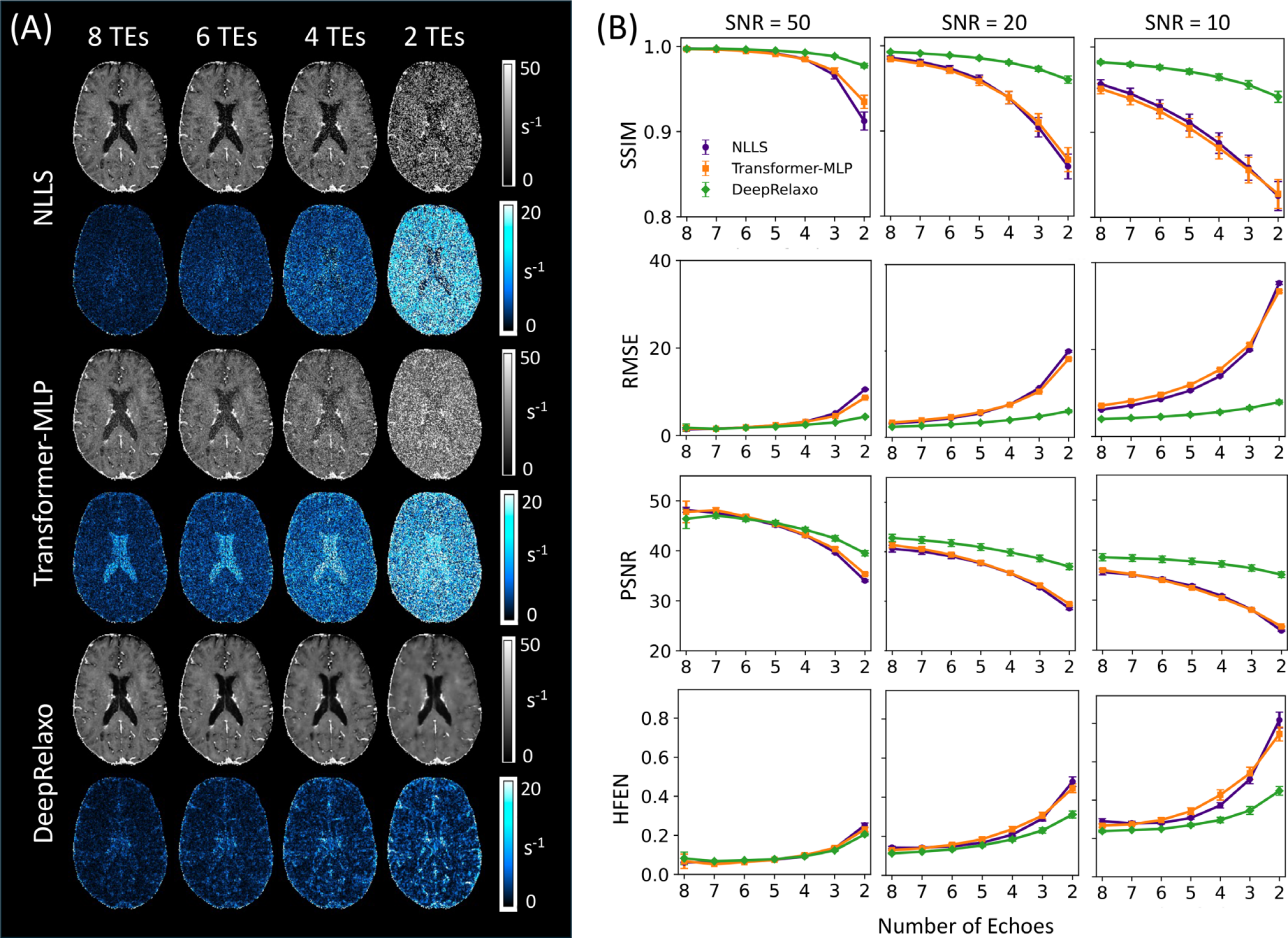
(A) Mid‐brain axial R2* maps reconstructed from simulated ME‐GRE sequences of different number of echoes (TEs: [3.4, 6.9, 10.4, 13.9, 17.4, 20.9, 24.4, 27.9] ms) at SNR = 20 using NLLS, Transformer‐MLP, and DeepRelaxo. The number of echoes is progressively reduced by discarding longer echoes. Each pair of rows displays the reconstructed R2* maps (top) and corresponding error maps (bottom) for each method. Error maps are computed as voxel‐wise differences between inferred R2* maps and the simulated ground‐truth R2* maps. (B) Quantitative comparison of R2* reconstruction across SNR levels as the number of echoes is reduced. SSIM, RMSE, PSNR, and HFEN are averaged over five simulated participants.

Qualitative comparisons at SNR 20 and 10 are shown in Figure 2A and Figure S4. At SNR 20, both NLLS and Transformer‐MLP lose R2* contrast when reduced to four or fewer echoes, with Transformer‐MLP notably overestimating low R2* values in CSF. In contrast, DeepRelaxo maintains consistent reconstruction quality even with only two echoes, with mild smoothing. At more challenging SNR 10, DeepRelaxo continues to produce meaningful R2* maps but with noticeable smoothing at short echoes, whereas the other two methods collapse into noise or signal dropout. Error maps relative to the simulated ground truth further demonstrate DeepRelaxo's robustness, revealing lower residuals and improved preservation of anatomical structures across varying noise levels and echo reductions.

Quantitative results at SNR = 20 are shown in Figure 2B that as the number of echoes decreases from 8 to 2, DeepRelaxo consistently achieves the best performance across all four metrics. HFEN increases from 0.11 to 0.31, compared with 0.14 to 0.48 for NLLS and 0.13 to 0.45 for Transformer‐MLP. SSIM decreases from 0.995 to 0.951 for DeepRelaxo, while larger reductions are observed for NLLS (0.992 to 0.862) and Transformer‐MLP (0.991 to 0.846). PSNR for DeepRelaxo decreases from 42.4 dB to 36.5 dB, whereas Transformer‐MLP and NLLS fall to 29.2 dB and 28.3 dB, respectively. RMSE increases from 2.03 to 7.20 s^−1^ for DeepRelaxo, compared with substantially larger increases for NLLS (2.32 to 18.61 s^−1^) and Transformer‐MLP (2.35 to 25.49 s^−1^). The same trends are observed at SNR = 10, with all methods degrading as echoes are reduced; however, the performance gap widens, as NLLS and Transformer‐MLP exhibit more rapid loss of structural fidelity, while DeepRelaxo remains comparatively consistent across echo reductions.

### 3.2 In Vivo Experiments

**FIGURE 3:**
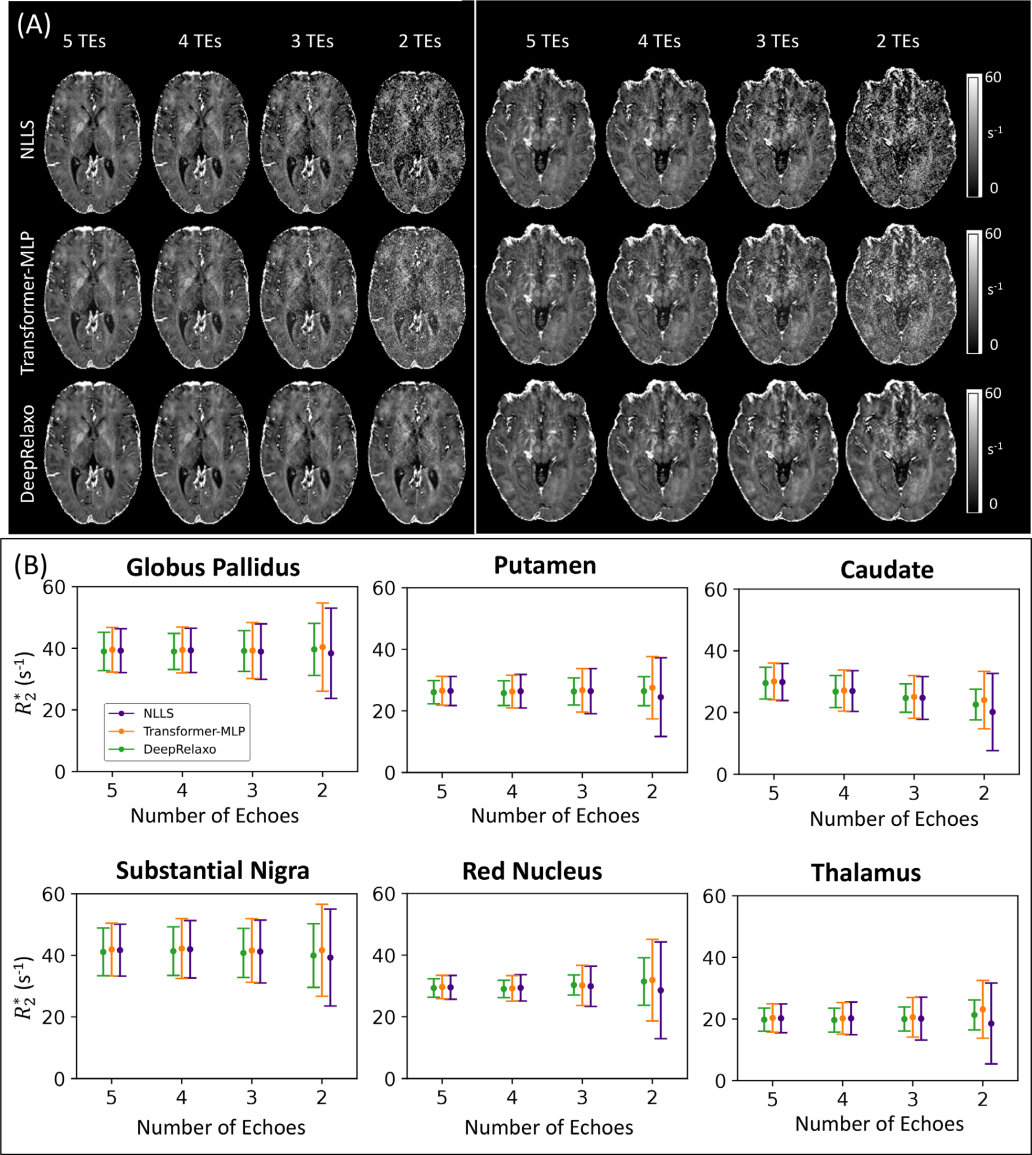
(A) Axial R2* reconstructions of two brain locations from a 5‐echo (TEs: [4.9, 9.9, 14.8, 19.8, 24.7] ms) in vivo ME‐GRE acquisition using NLLS, Transformer‐MLP, and DeepRelaxo, shown with decreasing number of echoes from [5 to 2]. (B) Mean ± SD of R2* values for the deep gray matter 2D ROIs across echo configurations.

In vivo results from Participant #1 at 3 T are shown in Figure 3A, where both NLLS and Transformer‐MLP exhibit progressive loss of anatomical clarity and tissue contrast as echoes are reduced, with severe degradation at two echoes. DeepRelaxo, by contrast, preserves structural detail and visual consistency, showing only mild smoothing at the lowest echo setting. ROI analysis on iron‐rich deep gray matter regions (Putamen, Caudate, Globus Pallidus, Thalamus, Red Nucleus, Substantia Nigra, as drawn in Figure S4) further supports these observations (Figure 3B). While all methods perform similarly with five and more echoes, NLLS and Transformer‐MLP show increasing variability as echoes are reduced below five, whereas DeepRelaxo maintains the lowest standard deviations, reflecting greater stability in these regions.

Results from two additional 3 T in vivo experiments using a different set of acquisition parameters (Participant #2 and Reproducibility participant 27‐year‐old female) are presented in Figures S6 and S7. DeepRelaxo demonstrates consistently strong performance, particularly for shortened echo trains, compared with the baseline methods.

**FIGURE 4:**
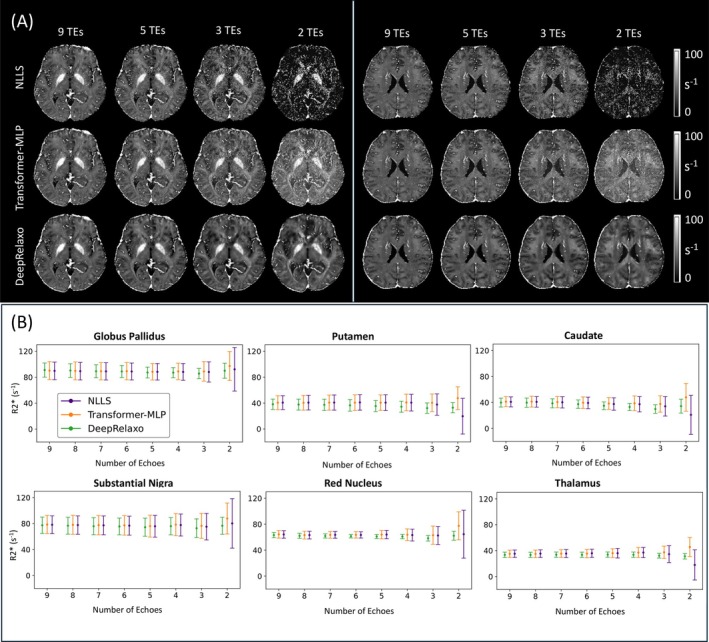
(A) R2* maps from two representative axial slices of mid brain reconstructed from a 9‐echo (TEs are: [5.1, 7.1, 9.2, 11.2, 13.3, 15.3, 17.3, 19.4, 21.4] ms) in vivo ME‐GRE acquisition at 7 T using NLLS, Transformer‐MLP, and DeepRelaxo. Reconstructions are shown as the number of available echoes decreases from 9 to 2. (B) Mean ± SD of R2* values of the deep gray matter 2D ROIs across echo configurations.

The 7 T result from Participant #3, as shown in Figure 4, demonstrates the same trend as the 3 T results. DeepRelaxo continues to produce consistent R2* maps across echo reductions with low variance and minimal bias relative to the full‐echo reference, while preserving anatomical contrast, whereas NLLS and Transformer‐MLP become increasingly noisy and structurally degraded. For example, at two echoes, in the Thalamus, DeepRelaxo achieves a standard deviation of 4.2 s^−1^ compared with 23.3 s^−1^ (NLLS) and 14.6 s^−1^ (Transformer‐MLP).

**FIGURE 5:**
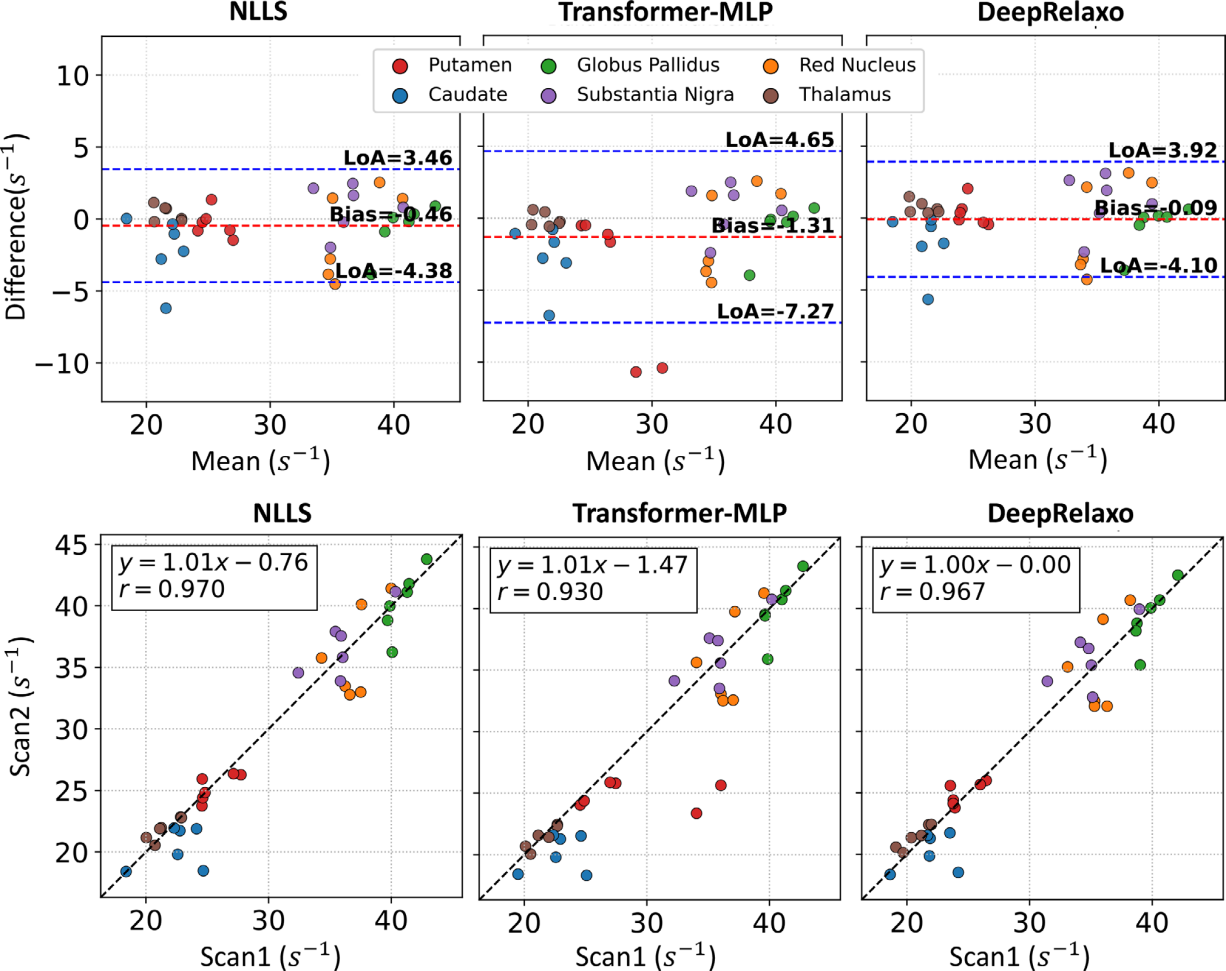
Bland–Altman and correlation plots comparing repeated scans with ROI‐wise R2* measurements across six deep‐gray‐matter regions from three participants. Each point corresponds to one ROI value. The solid line shows the mean bias, and the dashed lines indicate the 95% limits of agreement. The dashed diagonal line represents the line of identity.

The scan–rescan analysis in Figure 5, based on reconstruction results from the first four echoes, showed a small negative bias and moderate limits of agreement (LoA) for NLLS, whereas Transformer‐MLP exhibited a larger bias and wider LoA, indicating increased scan–rescan variability. In contrast, DeepRelaxo demonstrated near‐zero bias and comparatively tighter LoA. Pearson correlation analysis further indicated strong linear agreement for all methods (*r* ≥ 0.93). ROI‐wise statistical comparison of R2* estimates across deep gray matter structures from 10 in vivo scans is presented in Figure S8. Overall, ROI‐wise R2* measurements were largely consistent across methods. Although some regions showed statistically significant differences between methods, these differences were small (within 2.5%), indicating good practical agreement.

## 4 Discussion

Quantitative R2* mapping has long been limited by the need for long acquisitions and by reconstruction methods that are sensitive to noise and protocol variability. Traditional curve fitting (e.g., NLLS) is prone to bias from Rician noise, requires careful parameter initialization, and ignores spatial context. Recent deep learning methods improve robustness but typically assume fixed acquisition protocols. Their architectures are tied to a predetermined number of echoes, and their learned priors often fail to generalize when echo configurations differ across scanners, field strengths, or studies. This lack of flexibility remains a critical barrier to broader clinical translation.

In this work, we introduced DeepRelaxo, a cascaded framework that directly addresses these limitations. Its voxel‐wise Transformer‐MLP leverages a cross‐attention mechanism between echo times and signal magnitudes, allowing it to model arbitrary echo sequences without retraining for each protocol. This design breaks away from CNN‐based methods constrained to fixed input dimensions while also eliminating the need for parameter seeding or boundary conditions required by NLLS. Trained on synthetically generated data spanning a wide range of echo numbers, timings, and noise levels, the Transformer‐MLP provides robust, protocol‐agnostic R2* estimates.

To further enhance spatial consistency, DeepRelaxo adds a 3D U‐Net denoiser. This second stage learns local anatomical and noise patterns, refining the voxel‐wise estimates to yield clean and spatially coherent maps. The 3D U‐Net is especially critical in highly accelerated scenarios—such as few‐echo acquisitions—where voxel‐wise fitting alone struggles to recover meaningful structure. Together, the two modules combine temporal modeling of signal decay with spatial context for denoising, enabling accurate reconstructions even under challenging conditions.

Our results demonstrate that DeepRelaxo generalizes effectively across simulation and in vivo data at 3 T and 7 T. Reconstructions show consistent preservation of anatomical detail and noise suppression under echo reduction, while ROI analysis in iron‐rich deep gray matter confirms consistent mean values and lower variability compared with baselines. These regions are clinically important in studies of iron accumulation and neurodegenerative disease, highlighting the translational relevance of DeepRelaxo's robustness. Importantly, even in the most aggressive acceleration scenarios, DeepRelaxo continues to produce interpretable maps, whereas both NLLS and Transformer‐MLP degrade sharply.

Beyond robustness and generalizability, DeepRelaxo also offers high computational efficiency. Inference requires only seconds per 3D volume (e.g., 256 × 256 × 128) of multiple echoes (e.g., 8 GRE echoes) on a modern GPU, compared with several minutes for NLLS fitting. This near real‐time performance makes the method scalable to large datasets and well suited for time‐sensitive workflows.

There are, however, limitations. Due to memory constraints, the cascaded model was trained in two stages rather than end‐to‐end, which may limit optimal interaction between modules. Like other denoising approaches, DeepRelaxo faces a trade‐off between noise suppression and fine detail preservation at very low SNRs. The 3D U‐Net module, while effective, is tied to domain‐specific features and can introduce image contrast bias across field strengths, a limitation not observed in the Transformer‐MLP alone. This originates from the fact that R2* contrasts at different field strengths are different (i.e., domain shift). Synthetic training data improve generalizability but may not fully capture pathological variability, underscoring the need for further validation in patient populations. Finally, the current model focuses on mono‐exponential R_2_* mapping and does not explicitly account for multi‐component decay, subject motion, or macroscopic B_0_ field inhomogeneity in the synthetic signal generation. Further improvements may be achieved by incorporating realistic B_0_ inhomogeneity into the training data and extending the current magnitude‐only signal model to a complex‐valued formulation, which is beyond the scope of this Note.

## 5 Conclusion

In summary, DeepRelaxo provides a generalizable, self‐supervised solution for R2* mapping that overcomes the fixed‐protocol constraints of existing deep learning methods. By modeling arbitrary echo configurations and combining voxel‐wise temporal fitting with patch‐based spatial denoising, it achieves robust, accurate, and efficient reconstructions under echo‐accelerated and noisy acquisitions. These properties make DeepRelaxo a strong candidate for clinical translation, particularly in applications where short scan times and reliable deep gray matter quantification are essential.

## Funding

This work was supported by the National Health and Medical Research Council, 2030157. Australian Research Council, DE210101297, DP230101628.

## Supporting information


**Figure S1:** Transformer‐MLP Architecture.
**Figure S2:** 3D U‐Net Architecture.
**Figure S3:** Comparative DeepRelaxo R2* maps inferred from in vivo magnitude images without (top row) and with (bottom row) voxel spread function (VSF) correction(3) for Participant #2. Examples from slices near the nasal cavity and ear cavities, which are susceptible to macroscopic B_0_ field inhomogeneity, are shown. Arrows indicate regions affected by susceptibility variations where VSF preprocessing improves R2* estimation but not fully eliminated.
**Figure S4:** Mid‐brain axial R2* maps reconstructed from simulated ME‐GRE sequences of different number of echoes at SNR = 10 using NLLS, Transformer‐MLP, and DeepRelaxo. The number of echoes is progressively reduced by discarding longer echoes. Each pair of rows displays the reconstructed R2* maps (top) and corresponding error maps (bottom) for each method. Simulated TEs are [3.4, 6.9, 10.4, 13.9, 17.4, 20.9, 24.4, 27.9] ms.
**Figure S5:** Representative axial slices showing the deep gray matter regions segmented for ROI‐based evaluation in this study.
**Figure S6:** R2* maps from a representative axial slice of mid brain reconstructed from in vivo Participant #2 at 3 T using NLLS, Transformer‐MLP, and DeepRelaxo. Reconstructions are shown as the number of available echoes decreases from 9 to 2 for scan time reduction.
**Figure S7:** Mid‐brain axial R2* reconstructions from an in vivo participant (Reproducibility participant) using NLLS, Transformer‐MLP, and DeepRelaxo. Reconstructions are shown as the number of available echoes decreases from 8 to 2 for scan time reduction.
**Figure S8:** ROI‐wise statistical comparison of R2* estimates across deep gray matter structures over 10 in vivo scans. Boxplots show the distribution of ROI‐averaged R2* values, with individual scan measurements overlaid as dots. Central lines indicate medians, boxes represent interquartile ranges, and whiskers denote the full data range. Pairwise statistical comparisons between methods are indicated above each ROI (*p < 0.05; ns: not significant).
**Table S1:1:** Transformer–MLP ablation results at Echo = 8 (SNR = 20).
**Table S1:2:** Transformer–MLP ablation results at Echo = 4 (SNR = 20).
**Table S1:3:** Transformer–MLP ablation results at Echo = 2 (SNR = 20).
**Table S2:1:** 3D U‐Net denoiser depth ablation at Echo = 8 (SNR = 20).
**Table S2:2:** 3D U‐Net denoiser depth ablation at Echo = 4 (SNR = 20).
**Table S2:3:** 3D U‐Net denoiser depth ablation at Echo = 2 (SNR = 20).

## Data Availability

The data that support the findings of this study are available on request from the corresponding author. The data are not publicly available due to privacy or ethical restrictions. Codes are available at: https://github.com/sunhongfu/DeepRelaxo.
