# Supplementary material for: DeepRelaxo: Fast Mono‐Exponential Magnitude Brain R2* Mapping With Reduced Echoes Using Self‐Supervised Deep Learning
Source: Magn Reson Med. 2026 Apr 26;96(3):1293–302. doi: 10.1002/mrm.70405 (PMC13327502; doi:10.1002/mrm.70405)
Supplement: Supplementary file 1 — Figure S1: Transformer‐MLP Architecture. Figure S2: 3D U‐Net Architecture. Figure S3: Comparative DeepRelaxo R2* maps inferred from in vivo magnitude images without (top row) and with (bottom row) voxel spread function (VSF) correction(3) for Participant #2. Examples from slices near the nasal cavity and ear cavities, which are susceptible to macroscopic B0 field inhomogeneity, are shown. Arrows indicate regions affected by susceptibility variations where VSF preprocessing improves R2* estimation but not fully eliminated. Figure S4: Mid‐brain axial R2* maps reconstructed from simulated ME‐GRE sequences of different number of echoes at SNR = 10 using NLLS, Transformer‐MLP, and DeepRelaxo. The number of echoes is progressively reduced by discarding longer echoes. Each pair of rows displays the reconstructed R2* maps (top) and corresponding error maps (bottom) for each method. Simulated TEs are [3.4, 6.9, 10.4, 13.9, 17.4, 20.9, 24.4, 27.9] ms. Figure S5: Representative axial slices showing the deep gray matter regions segmented for ROI‐based evaluation in this study. Figure S6: R2* maps from a representative axial slice of mid brain reconstructed from in vivo Participant #2 at 3 T using NLLS, Transformer‐MLP, and DeepRelaxo. Reconstructions are shown as the number of available echoes decreases from 9 to 2 for scan time reduction. Figure S7: Mid‐brain axial R2* reconstructions from an in vivo participant (Reproducibility participant) using NLLS, Transformer‐MLP, and DeepRelaxo. Reconstructions are shown as the number of available echoes decreases from 8 to 2 for scan time reduction. Figure S8: ROI‐wise statistical comparison of R2* estimates across deep gray matter structures over 10 in vivo scans. Boxplots show the distribution of ROI‐averaged R2* values, with individual scan measurements overlaid as dots. Central lines indicate medians, boxes represent interquartile ranges, and whiskers denote the full data range. Pairwise statistical comparisons between methods are [file MRM-96-1293-s001.docx]

**Supplemental Figures**

**Figure S1:** Transformer-MLP Architecture.

Each scalar magnitude $M_{j}$ and echo time $t_{j}$ from the echo sequence is independently projected into a 256-dimensional embedding (a.k.a. token), producing sequences of embedded tokens $\boldsymbol{M}_{emb}$ and $\boldsymbol{t}_{emb}$. These token sequences are then processed by a two-layer Transformer decoder(1), with a 4-head cross-attention on $\boldsymbol{M}_{emb}$ and $\boldsymbol{t}_{emb}$, and then processed by a feed-forward network (hidden size 1024). The output of the first token is used as a global latent representation of the signal–time relationship. This token is passed to an MLP regression head comprising six fully connected layers (sizes: 1024, 512, 256, 128, 64, 1). Each hidden layer uses GELU activation, dropout (0.4), and layer normalization. The final output passes through a Softplus activation to ensure non-negative R2* estimates.

**Figure S2:** 3D U-Net Architecture.

As illustrated in Figure S2, 3D UNet(2) adopts a standard encoder–decoder architecture with skip connections. The encoder comprises four levels, each with two 3×3×3 convolutions (stride 1, padding 1) followed by batch normalization, SoftPlus, and 2×2×2 max pooling. Channel depth doubles from 32 to 256 across levels. The decoder symmetrically upsamples with trilinear interpolation, halves the channel depth at each level, and concatenates encoder features via skip connections. A final 1×1×1 convolution with Softplus produces the single-channel R2* map.

**Figure S3:** Comparative DeepRelaxo R2* maps inferred from in-vivo magnitude images without (top row) and with (bottom row) voxel spread function (VSF) correction(3) for Participant #2. Examples from slices near the nasal cavity and ear cavities, which are susceptible to macroscopic B_0_ field inhomogeneity, are shown. Arrows indicate regions affected by susceptibility variations where VSF preprocessing improves R2* estimation but not fully eliminated.

**Figure S4:** Mid-brain axial R2* maps reconstructed from simulated ME-GRE sequences of different number of echoes at SNR = 10 using NLLS, Transformer-MLP, and DeepRelaxo. The number of echoes is progressively reduced by discarding longer echoes. Each pair of rows displays the reconstructed R2* maps (top) and corresponding error maps (bottom) for each method. Simulated TEs are [3.4, 6.9, 10.4, 13.9, 17.4, 20.9, 24.4, 27.9] ms.

**Figure S5:** Representative axial slices showing the deep gray matter regions segmented for ROI-based evaluation in this study.

**Figure S6:** R2* maps from a representative axial slice of mid brain reconstructed from in vivo Participant #2 at 3T using NLLS, Transformer-MLP, and DeepRelaxo. Reconstructions are shown as the number of available echoes decreases from 9 to 2 for scan time reduction.

**Figure S7:** Mid-brain axial R2* reconstructions from an in vivo participant (Reproducibility participant) using NLLS, Transformer-MLP, and DeepRelaxo. Reconstructions are shown as the number of available echoes decreases from 8 to 2 for scan time reduction.

**Figure S8:** ROI-wise statistical comparison of R2* estimates across deep gray matter structures over 10 in-vivo scans. Boxplots show the distribution of ROI-averaged R2* values, with individual scan measurements overlaid as dots. Central lines indicate medians, boxes represent interquartile ranges, and whiskers denote the full data range. Pairwise statistical comparisons between methods are indicated above each ROI (*p < 0.05; ns: not significant).

**Supplemental Tables**

**Table S1.1:** Transformer–MLP ablation results at Echo = 8 (SNR = 20)

| **Architecture** | **HFEN ↓** | **PSNR (dB) ↑** | **SSIM ↑** | **RMSE ↓** |
| --- | --- | --- | --- | --- |
| Transformer-MLP  (128 / 512) | 0.3177 ± 0.0401 | 34.318 ± 0.540 | 0.9718 ± 0.0028 | 7.221 ± 0.196 |
| **Transformer-MLP**  **(256 / 1024) (ours)** | **0.1290 ± 0.0031** | **41.336 ± 0.525** | **0.9842 ± 0.0020** | **3.407 ± 0.066** |
| Transformer-MLP  (1024 / 4096) | 0.2724 ± 0.0148 | 35.934 ± 0.236 | 0.9768 ± 0.0017 | 5.239 ± 0.208 |

**Table S1.2:** Transformer–MLP ablation results at Echo = 4 (SNR = 20)

| **Architecture** | **HFEN ↓** | **PSNR (dB) ↑** | **SSIM ↑** | **RMSE ↓** |
| --- | --- | --- | --- | --- |
| Transformer-MLP  (128 / 512) | 0.2638 ± 0.0092 | 33.100 ± 0.209 | 0.9380 ± 0.0064 | 9.318 ± 0.085 |
| **Transformer-MLP**  **(256 / 1024) (ours)** | **0.2361 ± 0.0144** | **35.711 ± 0.422** | **0.9394 ± 0.0073** | **7.523 ± 0.114** |
| Transformer-MLP  (1024 / 4096) | 0.3368 ± 0.0285 | 32.060 ± 0.204 | 0.9328 ± 0.0062 | 8.879 ± 0.211 |

**Table S1.3:** Transformer–MLP ablation results at Echo = 2 (SNR = 20)

| **Architecture** | **HFEN ↓** | **PSNR (dB) ↑** | **SSIM ↑** | **RMSE ↓** |
| --- | --- | --- | --- | --- |
| Transformer-MLP  (128 / 512) | 0.4512 ± 0.0215 | 27.699 ± 0.288 | 0.8791 ± 0.0115 | 18.301 ± 0.221 |
| **Transformer-MLP**  **(256 / 1024) (ours)** | **0.4456 ± 0.0223** | **29.430 ± 0.223** | **0.8663 ± 0.0141** | **18.132 ± 0.257** |
| Transformer-MLP  (1024 / 4096) | 0.6178 ± 0.0461 | 25.448 ± 0.267 | 0.8609 ± 0.0126 | 22.813 ± 0.393 |

**Table S2.1:** 3D U-Net denoiser depth ablation at Echo = 8 (SNR = 20)

| **Architecture** | **HFEN ↓** | **PSNR (dB) ↑** | **SSIM ↑** | **RMSE ↓** |
| --- | --- | --- | --- | --- |
| DeepRelaxo  (U-Net depth 3) | 0.5272 ± 0.0301 | 31.37 ± 0.31 | 0.9238 ± 0.0076 | 12.29 ± 0.16 |
| **DeepRelaxo**  **(U-Net depth 4)** | **0.1124 ± 0.0031** | **42.78 ± 0.72** | **0.9928 ± 0.0008** | **2.40 ± 0.10** |

**Table S2.2:** 3D U-Net denoiser depth ablation at Echo = 4 (SNR = 20)

| **Architecture** | **HFEN ↓** | **PSNR (dB) ↑** | **SSIM ↑** | **RMSE ↓** |
| --- | --- | --- | --- | --- |
| DeepRelaxo  (U-Net depth 3) | 0.4882 ± 0.0313 | 32.06 ± 0.20 | 0.9011 ± 0.0110 | 14.57 ± 0.47 |
| **DeepRelaxo**  **(U-Net depth 4)** | **0.1838 ± 0.0100** | **39.88 ± 0.70** | **0.9805 ± 0.0020** | **3.98 ± 0.20** |

**Table S2.3:** 3D U-Net denoiser depth ablation at Echo = 2 (SNR = 20)

| **Architecture** | **HFEN ↓** | **PSNR (dB) ↑** | **SSIM ↑** | **RMSE ↓** |
| --- | --- | --- | --- | --- |
| DeepRelaxo  (U-Net depth 3) | 0.5211 ± 0.0290 | 29.16 ± 0.31 | 0.8953 ± 0.0101 | 14.94 ± 0.45 |
| **DeepRelaxo**  **(U-Net depth 4)** | **0.3110 ± 0.0178** | **37.00 ± 0.62** | **0.9603 ± 0.0043** | **6.04 ± 0.35** |

**References**

1. Vaswani A, Shazeer N, Parmar N, Uszkoreit J, Jones L, Gomez AN, Kaiser Ł, Polosukhin I. Attention is all you need. Proceedings of the 31st International Conference on Neural Information Processing Systems. Long Beach, California, USA: Curran Associates Inc.; 2017. p 6000–6010.

2. Ronneberger O, Fischer P, Brox T. U-Net: Convolutional Networks for Biomedical Image Segmentation. Medical Image Computing and Computer-Assisted Intervention – MICCAI 2015; 2015; Cham. Springer International Publishing. p 234-241. (Medical Image Computing and Computer-Assisted Intervention – MICCAI 2015).

3. Yablonskiy DA, Sukstanskii AL, Luo J, Wang XQ. Voxel Spread Function Method for Correction of Magnetic Field Inhomogeneity Effects in Quantitative Gradient-Echo-Based MRI. Magnetic Resonance in Medicine 2013;70(5):1283-1292.
